# Are you threatening me? Development and validation of the Conflict Escalation Questionnaire

**DOI:** 10.3389/fpsyg.2023.1164990

**Published:** 2023-07-20

**Authors:** Miriam Nicole Scheppa-Lahyani, Dieter Zapf

**Affiliations:** Department of Psychology, Goethe University Frankfurt, Frankfurt, Germany

**Keywords:** conflict, bullying, escalation, workplace, validation, instrument

## Abstract

This study aimed to develop and validate an instrument for measuring conflict escalation based on Glasl's conflict escalation model, which can also be used for measuring bullying conflicts. The instrument should be applicable both as a self-assessment and as an interviewer-assessment. In the first study, a first set of items measuring the stages of Glasl's model was developed and validated in an independent cross-sectional sample of 154 participants who completed the self-assessment. In 142 cases, interviews were conducted, and thus self and interviewer-assessments could be compared. In a second study, the final set of items was cross-validated on a second independent cross-sectional sample. In total, 105 participants completed the self-assessment only and 114 were part of the interview study. Because Glasl's model is complex, scale validation was based on a combination of classical statistical validation procedures. Both studies indicate good validity of the new instrument and provide evidence for Glasl's conflict escalation model. As expected, conflict escalation was positively related to negative affect, irritation, and depression. Relationship conflict was more prevalent in more highly escalated conflicts as compared to lower escalated conflicts. Victims of workplace bullying were classified in high escalation levels and showed higher inferiority in conflict situations compared to non-victims with highly escalated conflicts. The present instrument can be used to assess qualitative differences in conflict escalation and thus complements existing instruments to measure conflicts. It is especially useful for practitioners, as they can assess conflict escalation more accurately and thus better choose the appropriate form of intervention.

## 1. Introduction

Interpersonal conflict is one of the most important work-related stressors (Spector and Bruk-Lee, 2008) and can have a negative impact on both the person involved (e.g., Spector and Jex, 1998) and the organization in which the conflict takes place (e.g., Spector and Jex, 1998; De Dreu and Weingart, 2003). Because of the far-reaching consequences of conflicts, there is a need for effective interventions. To choose an appropriate form of intervention, it is important to know how far a conflict has escalated (Glasl, 1982). In this context, some scholars in the conflict research domain have developed theoretical models of how conflicts escalate and have proposed a stepwise escalation (Glasl, 1982; Fisher and Keashly, 1990; Rubin et al., 1994). One of the often-cited models in research and practice is Glasl's (1982) conflict escalation model (Keashly et al., 2020). According to this model, conflicts escalate stepwise over three main phases, based on qualitative changes in overt behavior, patterns of interaction, perceptions, attitudes, and feelings.

Although conflict escalation and Glasl's (1982) model are discussed in detail theoretically (Keashly et al., 2020), there is little empirical research, because escalation is difficult to measure. First, theoretical and empirical studies point to an escalation process from task conflicts to relationship conflicts to bullying (e.g., Zapf and Gross, 2001; Leon-Perez et al., 2015), as implied in Glasl's (1982) model. However, conflicts have mostly been operationalized through the frequency of conflict behaviors representing items of a conflict scale. Most conflict scales are developed according to the rules of classical test theory, in which one tries to gain high reliability by estimating internal consistency (Spector, 1992). All conflict characteristics measured by the items of the conflict scale are indicators of the same latent construct, meaning that a higher frequency of conflict behaviors represents a higher escalation of the conflict (e.g., Jehn, 1995; Spector and Jex, 1998). In contrast, Glasl's (1982) model considers the severity of conflict by focusing on qualitative changes; the frequency of conflict behavior plays a minor role. Although existing conflict questionnaires have contributed to our knowledge, they do not capture the quality of conflict escalation. Furthermore, they do not measure bullying conflicts. It is important, especially in practice, to distinguish highly escalated conflicts from bullying. Moreover, most instruments were developed based on self-reports (e.g., Jehn, 1995). Conflict and bullying are difficult to observe, but it can be valuable in a consulting setting to assess conflicts from an interviewer's perspective, explore the situation more deeply, and thus elicit the appropriate form of intervention.

To overcome these voids, the aim of the current study was (a) to develop an instrument to measure qualitative differences in conflict escalation based on Glasl's (1982) conflict escalation model, (b) which can also be used for measuring bullying conflicts, and (c) which can be used both as a self-assessment and as an interviewer-assessment. For this purpose, we develop and examine a first set of items in a first independent cross-sectional sample according to standard development criteria, and cross-validate the final item set on a second independent cross-sectional sample. We provide evidence for the escalation model and criterion-related validity of the new instrument, which is useful for further research and practice.

## 2. Theoretical framework

Conflict escalation can be understood as an increase in conflict intensity and severity. Most conflict escalation theories assume a stepwise escalation that continues if there is no intervention. For example, Pruitt (1998) described conflict escalation as a continuous change in conflict tactics with increasing intensity, starting with requests, followed by demands, complaints, and angry statements, and ending with threats, harassment, and abuse. Fisher and Keashly (1990) postulated a four-stage escalation model starting with discussions, polarization, followed by segregation, and ending in destruction. Similarly, Rubin et al.'s (1994) model suggests a four-stage escalation from intensity-increasing tactics to the involvement of the reference group, followed by increasing time involvement, and ending with harm to the other parties. A more comprehensive approach was offered by Glasl (1982). The model is described in the following section, as it forms the basis for the instrument developed in this study.

### 2.1. Glasl's conflict escalation model

Glasl's (1982) conflict escalation model combines the aspects of other conflict theories (Fisher and Keashly, 1990; Rubin et al., 1994). The model was developed to explore the mechanisms behind conflict escalation in different contexts, for example, political, family, or workplace conflicts. The recommendations of conflict intervention refer particularly to conflicts that have arisen in situations where the parties have to coordinate and cooperate to achieve a common goal, such as in the workplace (Glasl, 1982).

**Figure 1:**
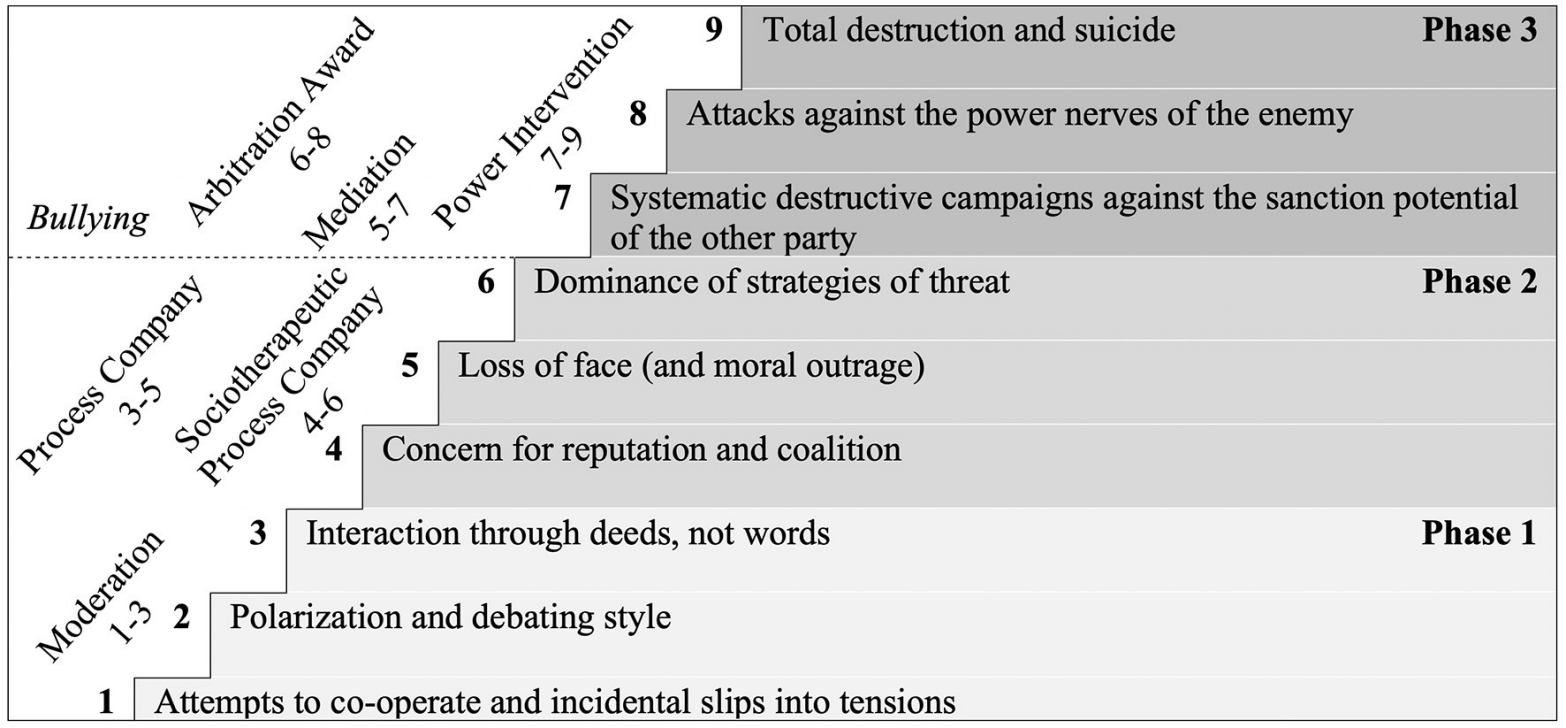
Glasl's (1982) conflict escalation model with intervention strategies. Figure adapted from Zapf and Gross (2001).

The model describes escalation as a stepwise process and assumes nine stages divided into three main phases (see Figure 1). The process begins with a first *phase* (“win-win” phase), in which factual disagreements about work occur (cf. task conflicts; Jehn, 1995). It is assumed that the workers have common interests and (overarching) goals. But disagreements arise about goals, plans, and assessments of facts and information relevant to the work. In this phase, the parties are sometimes cooperative and sometimes competitive. They want to demonstrate their superiority but do not want to completely dominate the other party. The best solution for a task or problem is sought and the parties communicate directly with each other.

When the conflict escalates into the *second phase* (“win-lose”), the conflict turns more to personal issues in which both parties polarize their positions and differences, and neglect the task (cf. relationship conflicts; Jehn, 1995). In this phase, it becomes clear that the protection of one's own self-esteem plays a major role in conflicts (Semmer, 2020). Having a positive view of oneself (personal self-esteem), but also being valued by significant others (social self-esteem) are fundamental human concerns (Alicke and Sedikides, 2009). Encountering relational devaluation, for example, by being derogated, excluded, or humiliated, implies a threat to self (Semmer, 2020). In the first phase, the feeling of being devalued may sometimes have been the (unconscious) reason for aggressive language. In contrast, in the second phase, the protection of one's own sense of self-worth comes to the fore, while the original factual problem loses importance. The norms of conversation are no longer respected. In this phase, there can only be one winner.

Finally, the conflict may escalate into the *final third phase* (“lose-lose”), which includes destructive behaviors, with the party with more power trying to destroy the reputation and self-esteem of the inferior party (like workplace bullying; Zapf and Gross, 2001). Communication is very difficult. Attempts are made to destroy and harm the other party, even if this means harming oneself. A constructive solution is no longer possible. The conflict can only be ended by a third party.

A detailed description of Glasl's (1982) conflict escalation model and its stages can be found in Appendix 1.

### 2.2. Difficulties in measuring conflict escalation

Although Glasl's (1982) model is used in research and practice quite frequently, there is hardly any empirical research on it. One reason for this is the lack of adequate instruments. A first attempt to operationalize Glasl's (1982) escalation model was made by Kolodej et al. (2005). They developed an instrument (in German: IKEAr), and the categorization of conflicts into specific conflict stages was based on a complex computer-based calculation, which is helpful for research but difficult to apply in practice.

Glasl's (1982) model is difficult to examine because it does not describe an easily definable construct. As mentioned earlier, conflicts are usually classified based on the frequency of certain conflict episodes, with more conflict episodes describing a stronger conflict (Jehn, 1995; Spector and Jex, 1998). In Glasl's (1982) model, however, this cannot be done in such a simplified way, as the individual stages have very specific and qualitatively different characteristics. For scale development, it would be ideal if each stage were characterized by certain items that do not occur in other stages. Alternatively, the characteristics of the escalation process could follow the Guttman scaling (Guttman, 1944). This would be the case if a characteristic of a particular stage is also present in all the stages below it, but not in the stages above it. However, it is not that easy. In Glasl's model, stages can be skipped. Also, some characteristics of a conflict of a particular stage may continue to occur at higher escalation levels while others may disappear which makes it difficult to apply Guttman scaling. The Glasl model is not very precise in his respect.

## 3. Study 1: validation of the Conflict Escalation Questionnaire

The first study aimed to generate items assessing the conflict stages consistent with Glasl's (1982) conflict escalation model and assess the reliability and validity of the scale. Detailed information on the scale development of the Conflict Escalation Questionnaire (CEQ) is reported in the methods section. In the following, we develop hypotheses for testing the construct validity of the CEQ.

### 3.1. Hypotheses development

Stress occurs when personally significant goals are threatened (Lazarus, 1999). Since the threat to personal interests and goals is part of many definitions of conflict (e.g., Thomas, 1992), conflicts can be classified as social stressors. The “Stress-as-Offense-to-Self' model (Semmer et al., 2019) focuses on the human need for good self-worth—in personal (personal self-worth) as well as in social terms (social self-worth). The protection and maintenance of self-worth are seen as the most significant goal (Sedikides and Alicke, 2019). The behavior of the other party in personalized conflicts is typically perceived as unfair and an attack on one's personal self-worth (Meier et al., 2013), and the more a conflict escalates, the more it is perceived as unfair. Escalation is characterized by increasingly intense emotional involvement (Glasl, 1982). The increasing threat to personal integrity is part of Glasl's conflict escalation model. Accordingly, we expected conflict escalation to be positively related to psychological strain.

According to stressor-strain models (e.g., Lazarus and Folkman, 1984), social stressors such as interpersonal conflicts are related to a variety of negative short-term outcomes such as anxiety, frustration, or anger (Spector and Jex, 1998; Ilies et al., 2011), and in the long term, to psychological and physical strains such as burnout, depression, or psychosomatic complaints (Spector and Jex, 1998; Spector et al., 2000). In line with Glasl (1982), relationship conflicts are more strongly associated with psychological health impairments as compared to task conflicts (De Dreu et al., 2004; Meier et al., 2013). To assess whether the developed instrument adequately measures conflict escalation, we assumed that the measured escalation is positively related to negative emotional reactions and psychological strain. To comprehensively consider these effects, taking into account stressor-strain frameworks (Lazarus and Folkman, 1984), and to circumvent random effects, the hypotheses were threefold.

First, we assumed that conflict escalation is positively related to negative affect. Negative affect encompasses a wide range of negative emotions, including, for example, anger, guilt, sadness, anxiety, and nervousness, and is positively related to interpersonal conflict (Spector and Bruk-Lee, 2008). Second, we expected the measured escalation to be positively related to irritation (Mohr, 1991; Dormann and Zapf, 2002). Irritation summarizes subjectively perceived emotional and cognitive strain and can be used to assess strain at work (Mohr et al., 2006). Third, we assumed that the measured escalation is positively related to depression. Depression can be defined as psychological strain and has consequently been associated with interpersonal conflict at work (Mohr, 1991; Spector and Jex, 1998; Dormann and Zapf, 1999, 2002). In sum, we hypothesize:

*Hypothesis 1*. Conflict escalation is positively related to negative affect.
*Hypothesis 2*. Conflict escalation is positively related to irritation.
*Hypothesis 3*. Conflict escalation is positively related to depression.

## 4. Method study 1

### 4.1. Sample and procedure

Participants were recruited via social networks in Germany. It was indicated that there should be a conflict at work that is current or has occurred in the last 6 months. Although the instrument is designed to classify current conflicts, past conflicts were also accepted for validation, as emotional events can be remembered relatively accurately over time (Yuille and Cutshall, 1986; Burke et al., 1992). For motivational reasons, participants had the opportunity to take part in a draw for vouchers.

Two types of surveys were conducted: online and interview surveys. At the beginning of both surveys, participants received a brief introduction and were informed that their participation was voluntary, anonymous, and confidential. Both survey methods included questions about the conflict and demographic variables. The online survey included only the self-assessment and was not accompanied by an interviewer. The interview survey covered both the interviewer-assessment, which was answered by the interviewer, and the self-assessment, which was responded to by the participant. We decided to include more than one interviewer in the study so that the results would be generalizable. The interviewers were persons from the university setting and were trained in the use of the instrument before the study began. A total of nine interviewers participated in Study 1.

Participants for Study 1 were recruited between March 2019 and February 2021. A total of 308 participants took part in the survey, of which 12 participants had to be excluded because they did not meet the study criteria. Of the remaining 296 participants, 154 completed the self-assessment only. In 142 cases, interviews were conducted and thus both assessments were conducted. Participants ranged in age from 19 to 63 years with a mean age of 36.03 years (*SD* = 12.16), and 68.03% of participants were female. The occupation was widely dispersed. Most participants (79.6%) were employed and 3.4% were self-employed. Most participants (27.21) had been working for between 5 and 10 years in the organization in which the conflict took place; 9.5% were working < 20 h per week and 29.3% more than 40 h per week. Moreover, 38.9% of participants described a current conflict; 45.1% had had a conflict with their supervisor, 48.8% with a colleague, and 6.1% with a subordinate.

### 4.2. Conflict Escalation Questionnaire

#### 4.2.1. Scale development

Based on the considerations regarding the difficulties mentioned above, instrument construction was inspired by Guttman scaling (Guttman, 1944) and behaviorally anchored rating scales (Schwab et al., 1975). We decided to develop separate subscales for each stage, which will be combined into a comprehensive escalation scale. The different subscales should consist of characteristics that are crucial for the respective stage. As it was crucial whether a certain behavior was shown during the conflict or not (“agree” vs. “disagree”), a dichotomous response scale was chosen.

The item pool is based on the guidelines proposed by Clark and Watson (1995), who recommended a clear conceptualization of the construct and the development of an overlapping item pool. For this purpose, the relevant characteristics of each stage were filtered out. The characteristics were clustered into emotions, feelings, attitudes, and behaviors. Since one aim of this study was to develop an instrument suitable for both self- and interviewer-assessment, the focus was on characteristics that were observable or described clear feelings such as trust. It turned out that the characteristics of stages 8 and 9 were very similar and that it would be difficult to distinguish between the two. Therefore, we decided to combine stages 8 and 9 into one common stage. Next, we decided whether a characteristic was a unique characteristic for the stage concerned, or whether it described a threshold. If it described a threshold, we assigned the aspect to the stage in which it occurred for the first or last time. After categorizing, the items were formulated. The self-assessment items were worded as “I,” while the interviewer-assessment items were worded as “the person.” To ensure comparability of the assessments, the items were identical in content. To circumvent social desirability, especially at higher stages when the behaviors become increasingly socially undesirable (e.g., threatening behavior), we worded the items as though the person had no choice but to behave in a socially undesirable way.

The first pool of items included a total of 64 items each for the self-assessment and the interviewer-assessment to measure the eight conflict stages. Since we had initially developed an overlapping item pool, we aimed to reduce the first set of items to those that were useful. For this purpose, reliability measures were considered (Kuder and Richardson, 1937; Cohen, 1960; Kelava and Moosbrugger, 2020).

#### 4.2.2. Classification of conflict

The items were used to classify the conflicts into the aforementioned stages, based on two assumptions of Glasl's (1982) model: (1) the characteristics of the stages are crucial for them, but not every characteristic has to occur, and (2) if only a minority of characteristics of higher stages occur, this can be a step toward escalation, but the conflict has not yet escalated to a higher stage. Only the co-occurrence of several characteristics initiates entry into the corresponding stage. Therefore, the percentage of characteristics that occurred per stage was calculated. Based on the two assumptions mentioned above, a stage was entered when at least 50% and therefore the majority of the respective characteristics occurred in the conflict. The highest stage that reached the 50% threshold was considered decisive. For example, if 3 out of 5 items of stage 5 were agreed (more than 50%) and < 50% of the items of stages 6, 7, or 8 were agreed, the conflict was classified as a stage 5 conflict. It is irrelevant how many characteristics of the lower stages occurred, as these can also be present in higher escalated conflicts.

#### 4.2.3. Final instrument

Our newly developed instrument is in German and measures conflict escalation based on Glasl's (1982) conflict escalation model. For this article, all items have been translated into English and have been checked by one native speaker. A back-translation was performed via DeepL and was checked again by the authors. Note that translation issues were not relevant to the conduct of the study, as the items were developed in German and used in a German-speaking sample.

Participants had to indicate on a 2-point response scale (“disagree” or “agree”) whether they experienced conflict-related aspects during the conflict period and thus, they had to decide whether a particular behavior had occurred. As the items mainly represent incidents that are either currently taking place or have taken place in the past, we decided against a multiple-response scale. Moreover, there was an additional response option “don't know” to find out if some items were difficult to understand or are not answered intentionally. The scale consists of two versions: self-assessment and interviewer-assessment. The first set of items consisted of 64 items each for the self-assessment and the interviewer-assessment measuring the eight stages. As a result of Study 1, the item set was reduced to a total of 35 items, with each stage measured by 3–5 items. The final item set is presented in Appendix 2. All final reliability values were acceptable to very good (see Table S7 in Appendix 3).

### 4.3. Measures

Measurement reliability was estimated using the coefficient omega (McDonald, 1999). For all measures, participants were asked to answer in relation to the conflict period.

#### 4.3.1. Negative affect

*Negative affect* was measured using a German translation (Breyer and Bluemke, 2016) of the Positive and Negative Affect Scale (PANAS) by Watson et al. (1988). Participants had to rate on a 5-point scale from “very slightly or not at all” to “extremely” how intensely they experienced emotions such as guilt (10 items). The scale had good internal consistency with ω above 0.85.

#### 4.3.2. Irritation

*Irritation* was measured with the German irritation scale by Mohr et al. (2005), which measures subjectively perceived emotional (5 items) and cognitive strain (3 items) in the work context. *Cognitive irritation* (e.g., “I have difficulty relaxing after work.”) describes a state in which the person cannot relax after work, while *emotional irritation* (e.g., “I get angry easily”) describes a state of agitated irritability. Response categories ranged from “strongly disagree” (=1) to “strongly agree” (=7). Internal consistency ω was good, with 0.93 for overall irritation.

#### 4.3.3. Depression

*Depression* was measured with a German depression scale developed by Mohr and Müller (2004). Participants had to rate on a 7-point scale from “never” to “almost every time” how often they experience symptoms of depression (eight items, e.g. “I see the future without hope”). Internal consistency was good with ω = 0.89.

### 4.4. Statistical analysis

Statistical analyses were conducted using the statistical software environment *R* (R Core Team, 2022, version 4.2.0). For the *evaluation of the items*, we have considered the following: (1) the relative frequency of missing values was calculated for each item. The response option “don't know” was treated as a missing value. Relative frequencies were standardized and compared at the 1% significance level (Grubbs, 1969) to determine if certain items were disproportionately unanswered. (2) To analyze whether the nominally scaled items are valid for both assessment conditions (participant and interviewer), Cohens κ (Cohen, 1960) was used to calculate interrater reliability. According to Landis and Koch (1977), κ-values below 0.40 are not acceptable, values between 0.40 and 0.60 are acceptable, values between 0.61 and 0.80 are good, and values above 0.80 are very good. (3) Item-total correlations indicating how highly an item correlates with the scale without that item were calculated; values above 0.40 are considered good and values above 0.30 are still acceptable (Field, 2018; Kelava and Moosbrugger, 2020). (4) Item difficulty was considered, indicating whether a particular item was answered “agree” or “disagree” particularly frequently, thus contributing little to the differentiation between individuals. Values between 0.20 and 0.80 are acceptable (Kelava and Moosbrugger, 2020). (5) Kuder–Richardson Formula 20 (*r*_*KR*20_,Kuder and Richardson, 1937), which is appropriate for dichotomous response scales, was calculated to measure the internal consistency of each stage after item selection. Values above 0.70 are good, while values above 0.60 are still acceptable (Cortina, 1993; Taber, 2018).

After classifying the conflicts into the respective stages based on the selected items, four criteria were considered to determine the *quality of the instrument*. (1) We investigated measurement errors. According to Glasl's (1982) model and with Guttman's (1944) scaling in mind, items at higher stages should not or only rarely be answered with “agree” compared to the classified stage. For this purpose, we examined how many items of higher stages were answered with “agree” on average compared to the classified stage. Thus, agreement on items describing a higher escalated stage than the assigned one represents a measurement error. Based on Glasl (1982), items of lower stages than the assigned one can be agreed to, but do not have to. For example, relationship conflicts can still be accompanied by task conflicts, but can also only be played out at the relationship level. (2) To assess agreement in the classification of conflicts between participant and interviewer, the interrater reliability was considered using Kendall's coefficient of concordance (Kendall's *W*; Kendall and Gibbons, 1990), which is an extension of Cohen's Kappa for multiple raters. It is particularly useful when the rating scale has a large range, as is the case here. (3) To examine the interrater agreement in more detail, the variance between participant- and interviewer-induced classifications was assessed. (4) To examine the criterion-related validity, Spearman's rank correlations (Spearman, 1904) were used to test the relationship of ordinal-scaled conflict escalation with negative affect, irritation, and depression predicted in Hypotheses 1–3.

## 5. Results: study 1

On average, each of the 26 variables measuring negative affect, irritation, and depression had 0.8% missing values (range: 0.7%−1.0%). As 5% or fewer missing values are considered inconsequential for analyses (Schafer, 1999), the number of missing values in this study was acceptable.

### 5.1. Item evaluation

Some items showed values above or under the acceptable range regarding the defined criteria mentioned above. These items were checked for their wording and whether the conflict characteristic addressed was also covered by another item with acceptable values. This was the case for all items with unacceptable values, so they were removed from the first set of items.

It turned out that there were problems with the items of stage 2, particularly in relation to internal consistency. There were two main reasons: first, the first half of the items for stage 2 was positively formulated and the other half was negatively formulated. Second, the positively formulated items described stage 2 characteristics, but could also be assigned to stage 1. This is because a key characteristic of stage 2 is that positive behaviors continue to be exhibited even though negative aspects occur at the same time. We decided to include the positively worded items of stage 2 in stage 1 and to measure stage 2 only with the negatively worded items. This procedure corresponds to the idea of Guttman scaling and is compatible with Glasl's escalation model: Features of the lower levels can continue to occur, but what is decisive are features that are newly added at the higher level.

Difficulties were also encountered at stage 6. A key characteristic of stage 6 is threats; all further actions and feelings build on this. However, it was found that items that addressed consequences of threats (e.g. “I feel pressured to make a decision by threats from the other party.”) did not perform well. Only one item that directly addressed the presence of threats met the criteria. Therefore, we decided to measure stage 6 with only one item, which is justifiable as stage 6 is mainly characterized by threats.

After an initial pre-selection, the remaining items were checked again for item-total correlation and internal consistency. All values corresponded to the defined criteria. The final set of items in Study 1 consisted of 28 items. All final characteristic values were acceptable to very good (see Table S7 in Appendix 3).

### 5.2. Classification

Based on the selected items, the conflicts were classified into the respective stage. Table 1 shows the frequencies of conflicts per stage for both the self-assessment and the interviewer-assessment. There were 3.4% of the conflicts in the self-assessment that could not be assigned to any stage. In the interviewer assessment, 0.7% were not assigned to any stage.

**Table 1:** Frequency of classification to the stages in both assessments in Study 1.

	**None**	**S1**	**S2**	**S3**	**S4**	**S5**	**S6**	**S7**	**S8**
Self^a^	10	35	38	47	5	25	24	17	95
Interview^b^	1	27	25	26	4	9	8	5	37

^a^N = 296.

^b^N = 142.

S, stage.

### 5.3. Instrument evaluation

To investigate measurement errors according to the principles of Guttman scaling (Guttman, 1944), we examined how many items of higher stages were answered with “agree” on average compared to the classified stage (see Table 2). As expected, the average agreement was high for the items of the same stage as assigned, and decreased rapidly for the items of the following higher stages. For example, conflicts assigned to stage 1 agreed on average with 90% of the items related to stage 1 and with 0% of the items related to stage 2. The results indicated a low measurement error. Only in the case of conflicts classified into stages 4 and 7, a larger measurement error was shown due to positive responses at the next higher escalation stage.

**Table 2:** Average agreement for the items of the respective stages based on the self-assessment in Study 1.

**Classified stage**	**Items of S1**	**Items of S2**	**Items of S3**	**Items of S4**	**Items of S5**	**Items of S6**	**Items of S7**	**Items of S8**
S1	**0.90**	0.00	0.07	0.03	0.11	0.00	0.03	0.03
S2	0.52	**0.70**	0.14	0.08	0.15	0.00	0.02	0.06
S3	0.36	0.76	**0.62**	0.11	0.17	0.00	0.04	0.09
S4	0.27	0.80	0.45	**0.72**	0.28	0.00	0.00	0.00
S5	0.28	0.80	0.63	0.38	**0.66**	0.00	0.11	0.17
S6	0.42	0.77	0.49	0.42	0.43	**1.00**	0.18	0.15
S7	0.27	0.68	0.60	0.44	0.46	0.27	**0.73**	0.29
S8	0.14	0.88	0.78	0.56	0.71	0.38	0.53	**0.78**

N = 296.

S, stage.

Bold = corresponding stages.

Lower triangular: average agreement in relation to items of lower stages than the classified stage; upper triangular (gray): average agreement in relation to items of higher stages than the classified stage (measurement errors).

With regard to interrater reliability and thus the agreement between self-assessment and interviewer-assessment (*N* = 142), the results showed that in 54.2% of the cases, the classification of the stages matched perfectly. In 75.4%, it was under- or overestimated by a maximum of one stage, and in 85.9% by a maximum of two stages. The ratio of over- and underestimations was similar. The results indicated satisfactory consistency between raters with *W* = 0.88 (*p* < 0.01).

To test criterion-related validity, the correlations of the classified stages with the relevant scales were determined. As expected, stages were positively related to negative affect, irritation, and depression (see Table 3). Therefore, Hypotheses 1, 2, and 3 were supported by the data.

**Table 3:** Mean values, standard deviations, internal consistencies, and intercorrelations in Study 1.

	** $\bar{x}$ **	***SD***	**1**	**2**	**3**	**4**
1. Escalation stages	–	–	–			
2. Irritation (overall)	3.58	1.47	0.47^**^	(0.93)		
3. Negative affect	2.58	0.76	0.55^**^	0.59^**^	(0.85)	
4. Depression	2.85	1.20	0.35^**^	0.61^**^	0.55^**^	(0.89)

McDonald's ω in parentheses. Spearman rank correlations. N = 296.

^**^p < 0.01 (two-tailed).

## 6. Brief discussion study 1

Based on Glasl's (1982) conflict escalation model, we developed an instrument that measures the qualitative differences in the various stages of Glasl's conflict escalation ladder and that can be used both as a self-assessment and as an interviewer-assessment. Due to insufficient values, 36 items had to be removed from the first item pool of 64 items. Based on the reduced instrument, Hypotheses 1, 2, and 3 could be confirmed: the results showed good criterion-related validity for negative affect, irritation, and depression. Overall, the results provide evidence for good psychometric properties of the instrument. Nevertheless, the results also underpin the difficulties in measuring conflict escalation. This was particularly evident in the classification of stage 2 and stage 6. Therefore, a further study should cross-validate the instrument and focus on revising these two stages.

## 7. Study 2: cross-validation of the Conflict Escalation Questionnaire

To ensure the validity of the instrument, a second study was conducted with a new sample. As described in Study 1, the items measuring stages 1, 2, and 6 were revised. The items measuring stage 7 were also adjusted, as they had been greatly reduced in the first study. The final instrument consists of 35 items measuring eight stages. The final items are listed in Appendix 2. To establish comparability, the same evaluation and classification strategy was used as in Study 1.

### 7.1. Hypotheses development

To examine the construct validity of the instrument, we first analyzed the same hypotheses as in study 1 with regard to psychological strain:

*Hypothesis 4*. Conflict escalation is positively related to negative affect.
*Hypothesis 5*. Conflict escalation is positively related to irritation.
*Hypothesis 6*. Conflict escalation is positively related to depression.

Moreover, we analyzed additional hypotheses with regard to relationship conflicts and bullying.

#### 7.1.1. Conflict escalation and relationship conflicts

According to Glasl (1982), phase 1 conflicts tend to be task-related, whereas from phase 2 onward, the conflict becomes person-related. Therefore, we assumed that the frequency of perceived relationship conflicts is lower in conflicts classified in stages 1 to 3, compared to conflicts classified in stage 4 onward.

*Hypothesis 7*. The frequency of relationship conflicts is higher in conflicts classified in stage 4 or higher compared to conflicts classified in stage 3 or lower.

Although Glasl (1982) assumed that conflicts from phase 2 onwards are mainly person-related, he did not specify the further development of task-related conflicts. Although the focus is on the relationship of the conflict parties, it might be plausible to experience task conflicts as well. On the one hand, one could assume that people who have a relationship conflict avoid the conflict partners, cooperate less with them and therefore have fewer task conflicts. On the other hand, if the conflict partners are not separated, the disturbed relationship may lead to even more task conflicts. Task and relationship conflicts are positively related (De Dreu and Weingart, 2003). Since task-related conflicts and relationship conflicts can coexist and task conflicts can occur in any conflict phase, no hypotheses were formulated here in relation to task conflicts.

#### 7.1.2. Conflict escalation and bullying

Bullying can be understood as a highly escalated and unresolved conflict (Einarsen et al., 2020). Zapf and Gross (2001) suggested that bullying may develop at the boundary between phases 2 and 3 of Glasl's (1982) model, as both are dominated by the use of increasingly severe means to harm the other party. Therefore, we assumed that bullying conflicts will be classified into stages 7 or 8.

*Hypothesis 8*. Conflicts of victims of workplace bullying are classified into stages 7 or 8.

Workplace bullying and highly escalated conflicts are related constructs (Leymann, 1996; Zapf and Gross, 2001). A conflict in phase 3, however, does not automatically mean that it is bullying. If the conflict partners were equally strong and could defend themselves equally well, it would not be bullying (Einarsen et al., 2020). The crucial difference is perceived inferiority to the disadvantage of the victim. Therefore, conflicts classified in phase 3 may overlap with the perception of workplace bullying, but do not necessarily fulfill all the criteria of a bullying conflict (Einarsen et al., 2020). To be able to identify bullying cases with the newly developed instrument, perceived inferiority must also be considered. Therefore, we expected victims of workplace bullying to perceive a higher inferiority as compared to non-victims whose conflicts were classified into stages 7 or 8.

*Hypothesis 9*. Victims of workplace bullying perceive a higher inferiority compared to non-victims with conflicts classified in stages 7 or 8.

## 8. Method study 2

### 8.1. Sample and procedure

Participants in Study 2 were recruited between July 2021 and June 2022 in Germany and underwent the same procedure as those in Study 1. The interviews were conducted by 22 interviewers. A total of 228 participants answered the questionnaire completely of whom 9 participants had to be excluded because they did not meet the study criteria. Of the remaining 219 participants, 114 were part of the interview study and 105 filled out the self-assessment only. Participants ranged in age from 16 to 61 years with a mean age of 32.98 years (*SD* = 10.54) and came from a variety of occupations; 76.7% of the participants were female. Most participants (69.9%) were employed, and 5.5% were self-employed. Most participants (25.6%) had been working between 5 and 10 years in the organization in which the conflict took place; 17.4% were working < 20 h per week and 31.1% more than 40 h per week. Moreover, 32.9% of the participants described a current conflict, 40.6% had had a conflict with their supervisor, 52.1% with a colleague, and 7.3% with a subordinate.

### 8.2. Measures

As in Study 1, McDonald's (1999) ω was calculated to ensure internal consistency. Conflict escalation, irritation, negative affect, and depression were measured with the same scales as in Study 1.

*Relationship conflict* was measured using a subset of the Social Stressors in Organizations 2.0 instrument (SSO2; Holz, 2003) as used in Kern et al. (2021), which is a further development and adaption of Frese and Zapf's (1987) scale. The items required responses on a 5-point scale ranging from “does not apply at all” to “fully applies.” The scale (five items, e.g. “You have to deal with the arrogant behavior of your colleagues.”) had a good internal consistency with ω = 0.87.

*Bullying* was measured with a German translation of the 9-item Short Negative Acts Questionnaire (SNAQ; Notelaers et al., 2019). It measures how often, on a 5-point scale from “never” to “daily,” negative social acts (e.g., “being ignored or excluded”) have occurred in the last 6 months. The scale had a good internal consistency with ω above 0.89.

*Inferiority* was measured with a self-developed scale. The scale describes how much influence on the development of the conflict exists (6 items, e.g., “I have control over how the conflict develops.”). Participants had to rate on a 5-point scale from “does not apply at all” to “fully applies.” An English translation of the items is presented in Appendix 2. The scale had a good internal consistency (ω = 0.90). Means, standard deviations, internal consistencies, and intercorrelations of these variables are presented in Table 6.

### 8.3. Statistical analysis

Statistical analyses were conducted using the statistical software environment *R* (R Core Team, 2022, version 4.2.0). To ensure comparability, the evaluation of the item set and the quality of the instrument were examined as in Study 1. Hypothesis 7 was tested using analysis of variance (ANOVA) and subsequent *post hoc* analyses (Tukey's HSD). For Hypothesis 9, one-tailed *t*-tests for independent samples were used. Levene's test indicated equal variances [*F*_(1, 70)_ = 0.07, *p* > 0.05], so no statistical corrections were made.

## 9. Results: study 2

On average, each of the 40 variables measuring negative affect, irritation, depression, relationship conflict, and workplace bullying had 0.1% missing values (range: 0.0%−1.4%); therefore, no efforts were made to manage the missing values in this study (Schafer, 1999).

### 9.1. Descriptive analysis

Overall, the items showed satisfactory values (see Table S7 in Appendix 3). Based on the final items, the conflicts were classified into their respective stages. Table 4 shows the frequencies of the conflicts in the various stages of the self-assessment and interviewer-assessment.

**Table 4:** Frequency of classification to the stages in both assessments in Study 2.

	**None**	**S1**	**S2**	**S3**	**S4**	**S5**	**S6**	**S7**	**S8**
Self^a^	2	22	27	28	8	17	41	15	59
Interview^b^	0	18	22	17	1	7	19	3	27

^a^N = 219.

^b^N = 114.

S, stage.

### 9.2. Instrument evaluation

Measurement errors were investigated with the same procedure as in Study 1 (see Table 5). As in Study 1, the results indicated a low measurement error. In the case of conflicts classified into stage 7, there was a larger measurement error.

**Table 5:** Average agreement for the items of the respective stages based on the self-assessment in Study 2.

**Classified stage**	**Items of S1**	**Items of S2**	**Items of S3**	**Items of S4**	**Items of S5**	**Items of S6**	**Items of S7**	**Items of S8**
S1	**0.84**	0.09	0.07	0.04	0.05	0.06	0.02	0.05
S2	0.70	**0.84**	0.15	0.10	0.13	0.07	0.06	0.04
S3	0.34	0.76	**0.60**	0.14	0.16	0.06	0.14	0.12
S4	0.63	0.54	0.38	**0.68**	0.05	0.09	0.08	0.08
S5	0.16	0.84	0.76	0.29	**0.68**	0.15	0.25	0.22
S6	0.41	0.79	0.55	0.35	0.35	**0.60**	0.20	0.15
S7	0.27	0.71	0.73	0.41	0.55	0.45	**0.63**	0.27
S8	0.20	0.93	0.75	0.60	0.74	0.50	0.59	**0.77**

N = 219.

S, stage.

Bold, corresponding stages.

Lower triangular: average agreement in relation to items of lower stages than the classified stage; upper triangular (gray): average agreement in relation to items of higher stages than the classified stage (measurement errors).

With regard to the interrater reliability of the self-assessment and interviewer-assessment (*N* = 114), the results showed that in 52.6% of the cases, the classification of the stages matched perfectly. In 75.4%, it was under- or overestimated by a maximum of one stage, and in 85.1% by a maximum of two stages. The ratio of over- and underestimations was similar. The results indicated satisfactory consistency between raters with *W* = 0.89 (*p* < 0.01) for escalation stages.

Concerning validity, negative affect, irritation, and depression were positively related to conflict escalation (see Table 6), thus supporting Hypotheses 4, 5, and 6.

**Table 6:** Mean values, standard deviations, internal consistencies, and intercorrelations in Study 2.

	** $\bar{x}$ **	***SD***	**1**	**2**	**3**	**4**	**5**	**6**	**7**
1. Escalation stage	–	–	–						
2. Irritation (overall)	3.72	1.59	0.37^**^	(0.96)					
3. Negative affect	2.62	0.77	0.42^**^	0.49^**^	(0.84)				
4. Depression	2.98	1.36	0.32^**^	0.57^**^	0.55^**^	(0.92)			
5. Relationship conflict	3.02	1.07	0.59^**^	0.30^**^	0.47^**^	0.27^**^	(0.87)		
6. Bullying	1.86	0.81	0.62^**^	0.42^**^	0.39^**^	0.27^**^	0.61^**^	(0.89)	
7. Inferiority	3.15	0.81	−0.37^**^	−0.38^**^	−0.45^**^	−0.37^**^	−0.25^**^	−0.38^**^	(0.90)

McDonald's ω in parentheses. Spearman rank correlations. N = 219.

^**^p < 0.01 (two-tailed).

**Figure 2:**
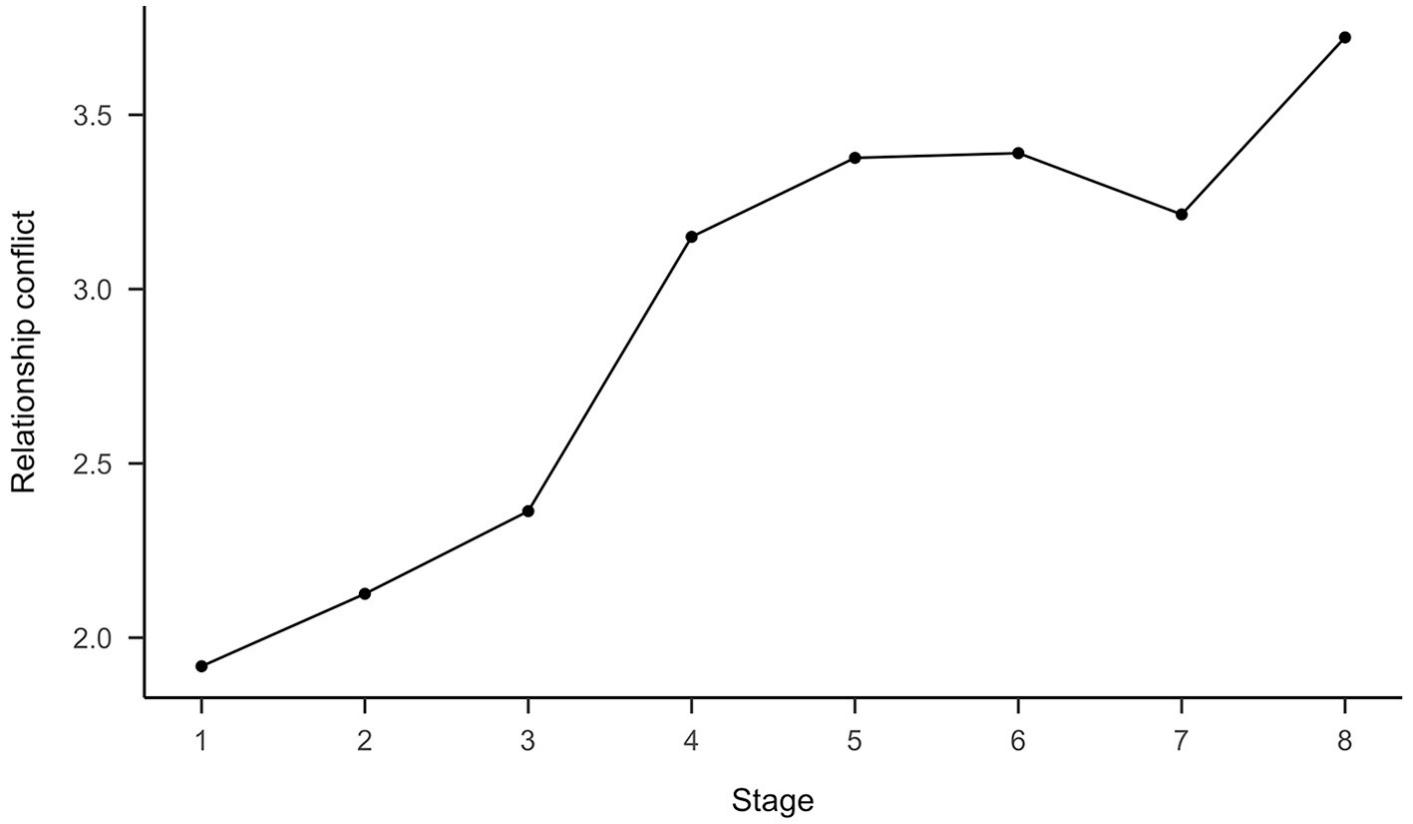
Changes in relationship conflict as a function of conflict stages. *N* = 219.

In relation to Hypothesis 7, relationship conflicts were positively related to conflict escalation (see Table 6). As shown in Figure 2, perceptions of relationship conflict increased across the stages, with a clear bend between stage 3 and stage 4. ANOVA revealed significant differences between the stages [*F*_(7, 206)_ = 19.54, *p* < 0.01] for relationship conflict. Further differences in the mean scores of relationship conflict for different stages were analyzed using *post hoc* analyses. The differences among stages 1, 2, and 3 were not significant (Tukey's HSD between 0.21 and 0.44, *p* > 0.05). Stage 1 was significantly different from stage 4 and higher stages (Tukey's HSD between 1.23 and 1.80, *p* < 0.05). Stage 2 was marginally different from stage 4 (Tukey's HSD = 1.02, *p* = 0.06) and significantly different from all higher stages (Tukey's HSD from 1.25 to 1.60, *p* < 0.01). Stage 3 showed no significant difference from stage 4 (Tukey's HSD = 0.79, *p* > 0.05), but did from all higher stages (Tukey's HSD between 0.85 and 1.36, *p* < 0.05). Therefore, Hypothesis 7 was largely supported by the data.

**Figure 3:**
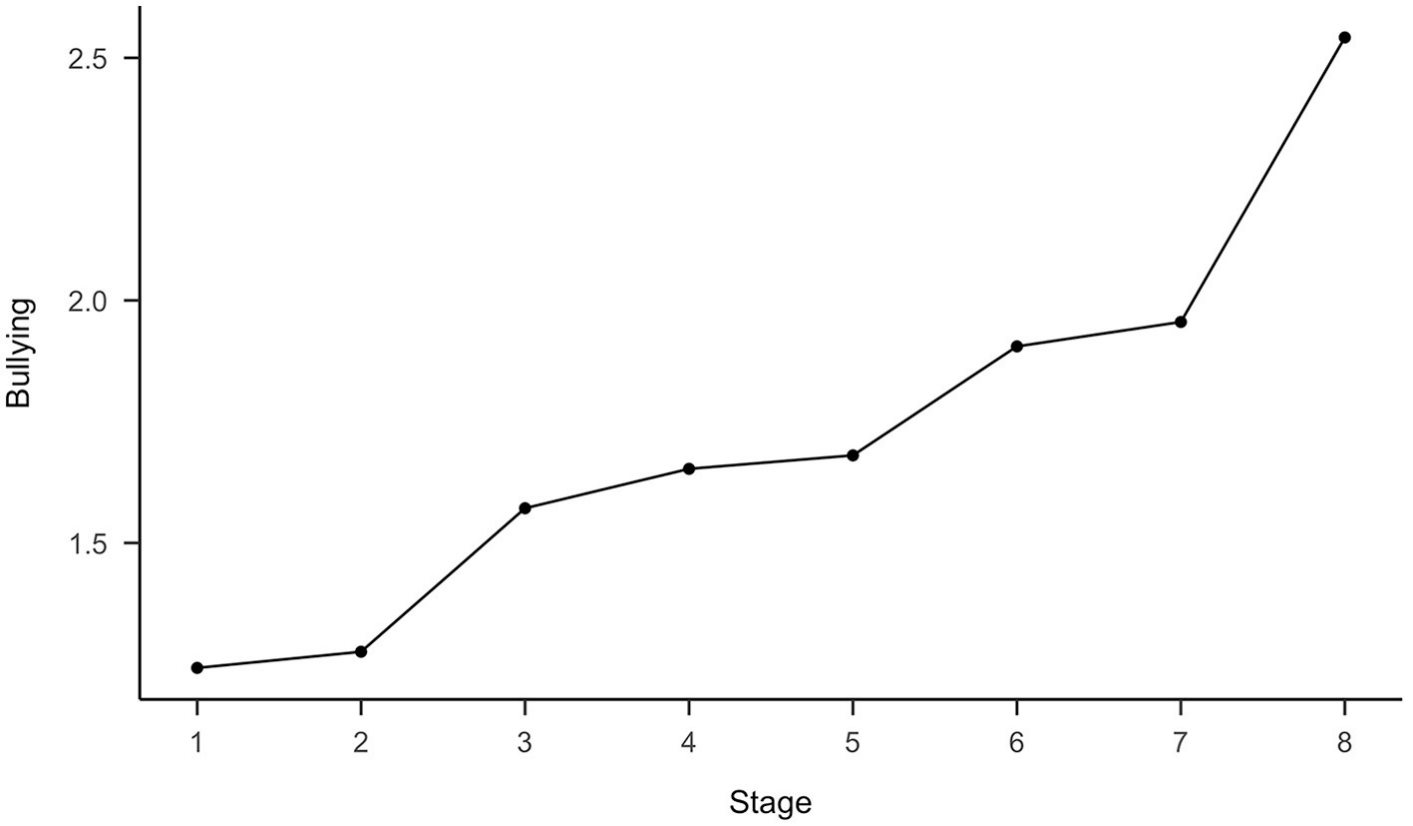
Changes in bullying as a function of conflict stages. *N* = 219.

With regard to Hypothesis 8, stages were positively related to bullying (see Table 6). As shown in Figure 3, perceptions of bullying increased across stages. A clear bend was noticeable between stage 7 and stage 8. In total, 5 participants were classified as having been exposed to workplace bullying (experiencing negative social acts at least weekly; Einarsen et al., 2020). Four bullying conflicts were classified in stage 8 and one in stage 6. Therefore, Hypothesis 8 was partially supported.

In Hypothesis 9, we expected victims of workplace bullying to experience higher inferiority, and thus less ability to influence the conflict, compared to participants whose conflicts were classified as at least stage 7 conflicts. As predicted, victims (*M* = 1.75, *SD* = 0.69, *N* = 4) differed significantly from participants classified in stages 7 and 8 (*M* = 2.80, *SD* = 0.79, *N* = 68) in terms of inferiority [*t*_(70)_ = 2.59, *p* < 0.01]. Therefore, Hypothesis 9 was supported by the data.

## 10. Brief discussion: study 2

In this study, we cross-validated an instrument that measures the escalation of conflicts at work based on Glasl's (1982) conflict escalation model and can be used as both a self-assessment and an interviewer-assessment. Overall, the results support the findings of Study 1 and provide evidence for good psychometric properties of the instrument. Good criterion-related validity was again achieved with negative affect, irritation, and depression. The assumption that the perceptions of relationship conflict are higher in phases 2 and 3 compared to phase 1 was also supported. Furthermore, the results indicate that the instrument can be useful for measuring bullying conflicts: the vast majority of bullying conflicts in this study were classified as highly escalated conflicts and victims differed significantly in their perceptions of inferiority as compared to participants whose conflicts were classified in stage 7 or 8. Accordingly, in practice, special attention should be paid to highly escalated conflicts that also show a high perception of inferiority to find the appropriate form of intervention.

## 11. General discussion

The present study aimed to develop and validate an instrument to assess conflict escalation according to Glasl's (1982) conflict escalation model that can be used as a self-assessment and interview-assessment and which can also help in the measurement of bullying conflicts. In doing so, we developed a new scale that captures the stages of Glasl's conflict escalation model. The new scale was tested in two independent cross-sectional samples and the results showed evidence of the validity of the model.

In support of Glasl's (1982) model, the results show that conflicts can be assigned to qualitatively different stages and that the different stages are separable. Only a small number of conflicts could not be assigned to any stage. There may be several reasons: first, a conflict may be at a very early stage of escalation and therefore exhibits just a few of the characteristic features, making it difficult to assign it. One may classify such situations as “no conflict.” Second, there may be other organizational conflicts that are not captured by Glasl's (1982) model; for example, non-task organizational conflicts such as conflicts relating to organizational culture or benefits (Bruk-Lee et al., 2013). Third, the model mainly discusses overt conflicts, but conflicts can also be indirect and subtle. In bullying conflicts especially, behavior is often indirect at the beginning (Keashly et al., 2020). Accordingly, future research should examine more closely which conflicts cannot be integrated into Glasl's (1982) model. Further items could be included in the instrument that also covers other forms of conflict.

Second, good concordance was found between self-assessment and interviewer-assessment. This adds to previous research in which conflicts and bullying were mostly documented through self-reports (e.g., Jehn, 1995; Notelaers et al., 2019). The results of this study suggest that conflicts can be adequately measured in an interview setting as well. However, some conflicts were under- or overestimated in their severity. This confirms that in some cases, it can be difficult to assess a conflict as a non-involved person. In the majority of cases, however, the assessments agreed, such that it can be assumed that the instrument can be used for both self-assessment and interviewer-assessment. It is recommended that the conflict should not only be observed but also discussed with the person concerned to gain sufficient insights.

Third, in support of the validity of the instrument relations with negative affect, irritation and depression were found in both studies. This is in line with common stress theories (Lazarus and Folkman, 1984) and previous studies (e.g., Dormann and Zapf, 1999, 2002; Spector and Bruk-Lee, 2008). Accordingly, the results support the validity of the instrument as well as Glasl's (1982) assumption that with increasing conflict intensity, negative emotions are also experienced to a greater extent.

Fourth, in line with our expectations, Study 2 shows that relationship conflicts play a major role in conflicts from stage 4 onwards, though it was shown that the frequency of relationship conflicts in stage 3 conflicts was not significantly different from the frequency of relationship conflicts in stage 4 conflicts. By and large, however, the results are consistent with Glasl's (1982) model, which assumes that conflicts become relationship-related from stage 4 onwards. Although no significant difference was found between stage 3 and stage 4 conflicts, the mean values differed in the expected direction. It should be noted that only a few conflicts were assigned to stage 4. Therefore, it is possible that the mean difference did not become significant due to the small sample size. In addition, some of the stage 3 conflicts may have already developed into relationship conflicts. Nevertheless, the results support the expected development of relationship conflict and thus support the model.

Fifth, as expected, bullying conflicts were classified as highly escalated conflicts (stages 7 or 8), which is in line with Zapf and Gross (2001) who located the occurrence of bullying between stages 6 and 7. One of the bullying cases was classified as stage 6 conflict, therefore still in line with our hypothesis. Furthermore, as expected, it could be shown that the perceived inferiority in the conflict was particularly high among victims as compared to non-victims. This is in line with the concept of bullying, as the main difference between bullying and highly escalated conflict is the inability to positively influence the conflict through one's own behaviors (Einarsen et al., 2020). Accordingly, the instrument can also be used to measure bullying conflicts. In practice, special attention should be paid to perceived inferiority in the conflict.

In sum, the instrument is an extension of existing conflict measures that measure conflict escalation via frequencies, such as those implied by Jehn (1995), and offers the possibility to examine conflicts in a more fine-grained way. This is particularly important in practice, as conflicts are often classified by subjective assessments. However, not every form of intervention is suitable for every level of conflict (Glasl, 1982), so an appropriate assessment is essential. The possibility of measuring the stages separately is useful for research and practice alike. It should be mentioned that the second study took place during the COVID-19 pandemic and data were collected during periods when severe restrictions prevailed in Germany, such as working full-time from home. Nevertheless, the results of Study 1 could be replicated and extended in Study 2, so that it can be assumed that the instrument could also be used for conflicts that play out in the absence of face-to-face encounters at the workplace.

### 11.1. Limitations and further research

Like any study, this study is subject to some limitations. First, due to the complexity of the model, no common test development procedures could be applied (e.g., Guttman, 1944), nor could the evaluation rely on common procedures such as factor analysis. Therefore, different strategies had to be combined to provide meaningful results. Although this is unusual and does not correspond to the common standard, the results are very promising. In particular, the use of behavioral anchors offers an advantage over the use of a Likert scale in terms of response bias (Furnham, 1986). It can therefore be assumed that the instrument is applicable. In future research, however, further studies should be conducted on how to improve the evaluation.

Second, both studies were cross-sectional. Although this was appropriate for the validation of the instrument, as the dependent variables should relate to the current state of the conflict (Glasl, 1982; Lazarus and Folkman, 1984), it might be interesting for further research to look more closely at the development of conflict escalation over time, for example, with weekly diary studies to analyze Glasl's (1982) assumed stepwise escalation. However, other courses of conflict escalation are also conceivable (Zapf and Gross, 2001), which could thus also be examined more closely.

Third, the periods over which the conflicts occurred vary in length, which may have influenced the results regarding criterion validity. For example, task conflicts tend to be of short duration, whereas bullying takes place over a longer period (e.g., 6 months). Accordingly, the impact of task conflict on mental health tends to be short-term, whereas longer-term and highly escalated conflicts tend to have long-term effects. Although initial findings on the relationship between conflict escalation and psychological strain were found in this study, additional variables should be examined in future research to differentiate short-term and long-term effects.

Fourth, the conflict situations were only described by one of the conflict parties. Accordingly, the perspective of the other party is missing. Conflicts depend on the individual's perception and can be differently assessed by the conflict parties. Accordingly, the conflict might have been perceived by the other party as not as severe or as even more highly escalated. Especially from an intervention perspective, it may be important to know the other side's views. However, this study was primarily concerned with developing an instrument that captures perceived escalation. Accordingly, considering both sides would be exciting for further research and practice, but was not crucial for this study.

Fifth, all data were collected from a German sample. The question is whether the conflict escalation model is culture-dependent and may only be applicable in Western countries. For example, conflict management strategies are used differently in collectivist cultures than in individualist cultures (Morris et al., 1998), so the process of escalation may be different. In particular, one can ask whether the separation of task-relatedness and social relation, which we believe is of some importance in Glasl's model, is universally applicable. In individualistic cultures, the individual self is seen as independent of the in-group (Markus and Kitayama, 1991). The individual has a functional attitude toward interpersonal relationships (Hofstede, 1980) and places personal achievement above personal position. People from individualistic cultures generally emphasize the need for autonomy, especially in regulating the flow of information (Hofstede, 1980). That is, team members are free to express their opinions and to offer task-related criticism even of statements made by more senior people.

In contrast, collectivism sees interpersonal relations as an end in itself (Triandis, 1995), and therefore places great emphasis on cultivating interdependence through fulfilling social norms and duties defined by relations established within the in-group (Hofstede, 1980). The dichotomy of individualism and collectivism is closely related to the dimension of power distance (Hofstede, 1980). In societies with low and medium power distance, status differences are not a fundamental obstacle to open communication. The detachment of personal status from the content and mode of communication makes direct negative feedback and explicit expressions of rejection or dissent possible which should be quite frequent in task-related conflicts (Merkin, 2006; Petersen and Zapf, 2023).

In collectivist societies with high power distance, on the other hand, pertinent issues cannot be expressed independently of personal status differences or obligations to others (Ting-Toomey, 1985). That is, both the content and the nature of an individual's communication depend on his or her status commitments to other group members. Direct issue-related statements can easily be perceived as a direct challenge to an individual's status and a threat to social face (Ting-Toomey and Kurogi, 1998). Against this background, it should be investigated whether Glasl's model, in which the lowest levels of escalation are still largely described in disregard of social relations, is also applicable to collectivist societies with high power distance.

### 11.2. Implications for practice

Our study has some important implications for practice. First, the questionnaire can be used in conflict or bullying support services to assess the severity of conflicts and thus be able to select the appropriate form of intervention (Glasl, 1982). It can also be used to distinguish a bullying conflict from a highly escalated conflict (Baillien et al., 2017). Second, the questionnaire can also be used in an organizational context, for example, in conflict management trainings, to better understand and raise awareness about conflict escalation. In this context, the organizational conflict culture must also be taken into account. The goals and values of the organization (Gelfand et al., 2012) can determine how conflicts are dealt with and thus escalate. Conflict management training has already proven its worth in this context (e.g., Zapf and Vartia, 2020). Our study also showed that in many cases, there were conflicts with supervisors, which has already been found in previous studies (see Zapf et al., 2020). Therefore, it can be particularly useful to train managers in understanding the mechanisms of conflict escalation.

## 12. Conclusion

This study adds to conflict research and practice by providing a new scale to assess conflict escalation according to Glasl's (1982) conflict escalation model. The validated instrument, which measures qualitatively different stages of conflict escalation, shows good psychometric properties and is relatively easy to apply. The instrument offers the possibility to assess conflict escalation through self-assessment and also through interviewer-assessment, and it may help to find the right form of conflict intervention.

## Data availability statement

The raw data supporting the conclusions of this article will be made available by the authors, without undue reservation.

## Ethics statement

Ethical review and approval was not required for the study on human participants in accordance with the local legislation and institutional requirements. The patients/participants provided their written informed consent to participate in this study.

## Author contributions

MS-L and DZ conceived and designed the study and contributed in the interpretation of results. MS-L was responsible for data collection, analyzed the data, and wrote the first original draft. DZ was involved in reviewing and editing of the manuscript. All authors read and approved the final manuscript.
