# Supplementary material for: Are you threatening me? Development and validation of the Conflict Escalation Questionnaire
Source: Front Psychol. 2023 Jul 20;14:1164990. doi: 10.3389/fpsyg.2023.1164990 (PMC10399688; doi:10.3389/fpsyg.2023.1164990)
Supplement: Supplementary file 1 [file Data_Sheet_1.pdf]

Appendix 1

Glasl's conflict escalation model

The model describes escalation as a stepwise process and assumes nine stages divided into three main phases (see Figure 1). The process begins with a first phase ("win-win" phase) in which factual disagreements about work occur (cf. task conflicts; Jehn, 1995). It is assumed that the workers have common interests and (overarching) goals. But disagreements arise about goals, plans, assessments of facts and information relevant to the work. In this phase, the parties are sometimes cooperative, sometimes competitive. They want to demonstrate their superiority but do not want to completely dominate the other party. The best solution for a task or problem is sought and the parties communicate directly with each other. The phase comprises stages 1 to 3. *Stage 1* is described as attempts at cooperation and occasional tensions. In this stage there are disagreements about goals, procedures or evaluation of solutions. Occasionally, tensions arise because people interrupt each other, become loud, sarcastic or even aggressive. However, this behaviour is basically regretted, because one is interested in a common solution. The longer these disagreements last, the more often occasional tensions occur. The parties are, however, still convinced that the confrontations can be resolved over time through rational discussion. *Stage 2* is the stage of polarization and debating style. The parties have experienced discussions no longer being productive, so they start to debate. This means that less and less attention is paid to the arguments of the other party and one increasingly comes to the conclusion that the other party simply does not understand one's own good arguments. Hope sinks that the other party can be convinced. Impressing the other party with better arguments becomes more and more important, while the arguments of the other side go unheard. At *stage 3*, deeds, not words, are the main characteristic. With their actions, the parties want to influence the other party's opinion and behaviour. The parties are increasingly realising

that the other party is not showing any insight. Thus, words no longer lead anywhere. Therefore, action must be taken and facts must be created. At the latest, the other side should realise that their position was wrong. Since the parties talk little to each other, misunderstandings and misinterpretations arise. The parties increasingly expect negative actions from the other party and empathy visibly dwindles. Empathy for the other person gives way to mistrust and negative expectations, which further exacerbates the conflict. Nevertheless, the parties still know that the problem can only be solved if they go through a solution process together.

When the conflict escalates into the second phase ("win-lose"), the conflict turns more to personal issues in which both parties polarize their positions and differences, and neglect the task (cf. relationship conflicts; Jehn, 1995). In this phase it becomes clear that the protection of one's own self-esteem plays a major role in conflicts (Semmer, 2020). Having a positive view of oneself (personal self-esteem), but also being valued by significant others (social self-esteem) are fundamental human concerns (Alicke and Sedikides, 2009). Encountering relational devaluation, for example, by being derogated, excluded or humiliated, implies a threat to self (Semmer, 2020). While in the first phase the feeling of being devalued may sometimes have been the (unconscious) reason for aggressive language, in the second phase the protection of one's own sense of self-worth comes to the fore, while the original factual problem loses importance. The norms of conversation are no longer respected. In this phase there can only be one winner. The phase comprises stages 4 to 6. In *stage 4*, due to their need to protect their selves, the parties are now concerned with reputation and coalition. The opponents look for supporters and allies, parties are formed and nasty rumours are spread about the other party. It is no longer about the original cause, but about winning the conflict. The conflict behaviour is now purely competitive and direct communication decreases more and more. The aim is to blame and to denigrate the other party, usually in public. In *stage 5*, the social identity of the parties has

been attacked several times, so that a "loss of face" is the result. Mutual attacks become more direct and personal, immoral "punches below the belt" begin. Wherever possible, one wants to expose the opponent. The loss of morale and mutual trust goes hand in hand with the loss of face. The mere sight of the opponent generates negative feelings. Effective communication is no longer possible. In *stage 6*, threatening strategies are used. The resistance of the other party is no longer accepted and must be broken. The conversation is dominated by threats and counter-threats, threatening gestures and threatened sanctions.

Finally, the conflict may escalate into the *final* third phase ("lose-lose"), which includes destructive behaviours, with the party with more power trying to destroy the reputation and self-esteem of the inferior party (like workplace bullying; Zapf and Gross, 2001). Communication is very difficult. Attempts are made to destroy and harm the other party, even if this means harming oneself. A constructive solution is no longer possible. The conflict can only be ended by a third party. This phase comprises stages 7 to 9. In *stage 7*, the parties start systematic destructive campaigns against the sanction potential of the other party. They attempt to harm each other, with actions directed against work-related objects (e.g., deleting important files). The parties also accept self-related damage as long as the other party perceives greater damage. *Stage 8* is characterized by attacks on the opponent's power nerves. The goal is to destroy the power and existence of the other party. Violence is used to achieve this goal, but not in a life-threatening way. At *stage 9*, the parties end in total destruction and suicide. All possible power and violence is used to destroy the other party. Suicide is acceptable to both parties if they know that it will destroy the other party as well.

Appendix 2

Conflict Escalation Questionnaire (CEQ) – Self-assessment

Instructions: Please indicate below whether the statements mentioned apply to the conflict situation. You should relate the statements only to the conflict and not to how you act, think or feel in general. Some points refer to your behaviour, but also to the behaviour of the other party.

Response scale: "disagree" (= 0) or "agree" (= 1)

Stage	Item	Number of items that must be answered with "agree" to enter the particular escalation stage (50% criterion)
S1	1.1: Each of us tries to find a compromise to avoid an increase in tensions.	2 out of 4
	1.2: I think that the tensions between the other party and I can be resolved if we talk to each other.	
	1.3: Each of us attempts to understand the other's point of view.	
	1.4: Each of us tries to find a joint solution that is acceptable to both parties.	
S2	2.1: At least one of us is convinced of the correctness of their point of view and is therefore no longer willing to readily give it up.	2 out of 3

	2.2: At least one of us insists on their opinion in our disputes, having the conviction that it is best for all.	
	2.3: At least one of us sees ourselves in the superior position.	
S3	3.1: At time conversations are broken off because they lead nowhere.	2 out of 4
	3.2: I increasingly have the impression that conversations are pointless because it has become clear that the other party cannot be convinced to change their mind.	
	3.3: The conflict has progressed to a point where the other party and I increasingly see ourselves as competitors.	
	3.4: The disputes have shown that discussions no longer achieve anything. Instead, positions have to be moved by deeds.	
S4	4.1: It comes to "punitive measures" such as provoking, ignoring, disrespecting, etc.	3 out of 5
	4.2: I have the impression that everything the other party does is only to provoke me.	
	4.3: Provocations take place when there are no witnesses.	
	4.4: I have the feeling that the conflict is becoming increasingly subjective.	
	4.5: The conflict is dominated by personal attacks.	
S5	5.1: The conflict has resulted in damage of my, and/or the other party's reputation.	3 out of 5

	5.2: The conflict has progressed to the point where at least one of us has been humiliated or belittled.	
	5.3: I feel uncomfortable when the other party enters the room.	
	5.4: The conflict has escalated to the point where at least one of us no longer has favorable intentions towards the other.	
	5.5: I feel that I cannot trust the other party.	
S6	6.1: Serious threats have been expressed.	2 out of 4
	6.2: I feel pressured by the other party.	
	6.3: I feel that my freedom of action is severely restricted by the other party.	
	6.4: At least one of us is behaving very aggressively.	
S7	7.1: My thoughts increasingly revolve around how to secure my professional existence.	3 out of 5
	7.2: Because of the conflict, the other party and I lie and mislead.	
	7.3: The conflict has led to my job or the job of the other party being at stake.	
	7.4: At least one of us acts unscrupulously and callously in the conflict.	
	7.5: The conflict has escalated to the point where it has become impossible to find a good solution.	

S8	8.1: The conflict burdens me so much that I am considering leaving the organisation.	3 out of 5
	8.2: The conflict has escalated to such an extent that I can no longer imagine working with the other party at all.	
	8.3: The conflict costs so much energy and time that I tend to neglect my work.	
	8.4: I think that the conflict can only be ended if one of us leaves the organisation/department.	
	8.5: I will no longer work with the other party, no matter the cost.	

Scoring Instructions:

The scale measures the escalation of conflict according to Glasl's conflict escalation model and ranges from 0 to 8.

0 means no conflict, 1-8 corresponds to the escalation stages according to Glasl, 8 is the highest escalation stage and combines stages 8 and 9.

In order to determine the conflict level, two steps must be followed:

Step 1: Note all stages in which the 50% criterion was reached (50% of the items of the respective stage were answered with "agree").

Step 2: The highest stage in which the 50% criterion was reached describes the given escalation stage. If none of the stages have exceeded 50%, the scale value is set to 0.

Inferiority Scale

Response scale: “does not apply at all” (= 1) to “fully applies” (= 5)

1. I feel in an inferior position. (Reversed item)

2. I am able to defend myself.

3. I have control over how the conflict develops.

4. I have an influence on how the conflict develops.

5. I can determine how the conflict continues.

6. I have the chance to influence the conflict positively.

Appendix 3

Table 7

Values of the selected items in Study 1 and Study 2 based on the self-assessment

		Study 1[a]					Study 2[c]				
Stage	Item	$r_{i(t-i)}$	drop α	*KR-20*	κ^b	*p*	$r_{i(t-i)}$	drop α	*KR-20*	κ^d	*p*
S1	1.1	.58***	.69	.77**	.44*	.37**	.58***	.67	.75**	.48*	.37**
	1.2	.54***	.75		.71**	.54**	.34**	.80		.61**	.54**
	1.3	.62***	.62		.60*	.35**	.59***	.66		.47*	.33**
	1.4						.69***	.61		.63**	.36**
S2	2.1	.58***		.74**	.41*	.68**	.58***	.46	.69*	.34	.78**
	2.2	.58***			.51*	.81*	.52***	.51		.40*	.70**
	2.3						.33**	.77		.66**	.76**
S3	3.1	.39**	.75	.75**	.67**	.64**	.41***	.65	.68*	.48*	.53**
	3.2	.65***	.62		.65**	.68**	.53***	.57		.68*	.67**
	3.3	.41***	.74		.69**	.37**	.35**	.69		.41*	.29**

	3.4	.64***	.62		.61**	.56**	.57***	.54		.68**	.65**
S4	4.1	.56***	.80	.82**	.68**	.50**	.50***	.69	.74**	.52*	.50**
	4.2	.60***	.78		.70**	.27**	.43***	.71		.47*	.25**
	4.3	.57***	.79		.59*	.28**	.46***	.70		.49*	.28**
	4.4	.61***	.78		.66**	.41**	.57***	.66		.44*	.47**
	4.5	.65***	.76		.67**	.25**	.54***	.68		.49*	.20**
S5	5.1	.55***	.75	.79**	.52*	.33**	.53***	.76	.79**	.52*	.41**
	5.2	.55***	.75		.69**	.36**	.51***	.77		.55*	.35**
	5.3	.55***	.76		.71**	.53**	.55***	.76		.57*	.47**
	5.4	.63***	.73		.61**	.30**	.66***	.72		.51*	.30**
	5.5	.53***	.76		.64**	.68**	.60***	.74		.60**	.49**
S6	6.1				.76**	.22**	.30**	.64	.63*	.70**	.09
	6.2						.51***	.59		.45*	.48**
	6.3						.49***	.50		.49*	.48**
	6.4						.37**	.48		.53*	.22**
S7	7.1	.61***	.67	.78**	.60*	.24**	.38**	.68	.70**	.59*	.24**

	7.2	.56***	.76		.73**	.34**	.45***	.65		.69**	.29**
	7.3	.62***	.67		.61**	.21**	.42***	.67		.39*	.20**
	7.4						.59***	.59		.63**	.41**
	7.5						.44***	.66		.55*	.33**
S8	8.1	.69***	.84	.87**	.72**	.44**	.56***	.71	.76**	.79**	.35**
	8.2	.82***	.81		.80***	.43**	.66***	.67		.83***	.37**
	8.3	.48***	.88		.55*	.21**	.31**	.79		.52*	.22**
	8.4	.77***	.83		.69**	.42**	.55***	.71		.74**	.35**
	8.5	.68***	.83		.67**	.24**	.59***	.70		.57*	.22**

Note. [a]$N = 296$, [b]$N = 142$, [c]$N = 219$, [d]$N = 114$, κ = Cohens Kappa, $r_{i(t-i)}$ = item-total correlation, p = item difficulty, *KR-20* = internal consistency, drop α = internal consistency, if an item is dropped.

*** = very good, ** = good, * = acceptable.
